# Predictors of Increased Daytime Sleepiness in Patients with Chronic Obstructive Pulmonary Disease: A Cross-Sectional Study

**DOI:** 10.1155/2016/1089196

**Published:** 2016-10-16

**Authors:** Claudia Enz, Stefanie Brighenti-Zogg, Esther Helen Steveling-Klein, Selina Dürr, Sabrina Maier, David Miedinger, Jörg Daniel Leuppi

**Affiliations:** ^1^University Clinic of Medicine, Cantonal Hospital Baselland, Liestal, Switzerland; ^2^Medical Faculty, University of Basel, Basel, Switzerland; ^3^Department of Health Sciences and Technology, ETH Zurich, Zurich, Switzerland

## Abstract

*Background*. Patients with Chronic Obstructive Pulmonary Disease (COPD) suffer from increased daytime sleepiness. The aim of this study was to identify potential predictors of subjective daytime sleepiness with special regard to sleep-related breathing disorder and nocturnal activity.* Methods*. COPD patients were recruited at the University Hospital Basel, Switzerland. COPD risk groups A–D were determined according to spirometry and COPD Assessment Test (CAT). Breathing disorder evaluation was performed with the ApneaLink device. Nocturnal energy expenditure was measured with the SenseWear Mini Armband. Subjective daytime sleepiness was recorded using the Epworth Sleepiness Scale (ESS).* Results*. Twenty-two patients (36%) were in COPD risk group A, 28 patients (45%) in risk group B, and 12 patients (19%) in risk groups C + D (*n* = 62). Eleven patients (18%) had a pathological ESS ≥ 10/24. ESS correlated positively with CAT (*r* = 0.386, *p* < 0.01) and inversely with age (*r* = −0.347, *p* < 0.01). In multiple linear regression age (*β* = −0.254, *p* < 0.05), AHI (*β* = 0.287, *p* < 0.05) and CAT score (*β* = 0.380, *p* < 0.01) were independent predictors of ESS, while nocturnal energy expenditure showed no significant association (*p* = 0.619).* Conclusion*. These findings provide evidence that daytime sleepiness in COPD patients may partly be attributable to nocturnal respiratory disturbances and it seems to mostly affect younger patients with more severe COPD symptoms.

## 1. Introduction

Excessive daytime sleepiness or hypersomnolence is a nonspecific but frequent symptom in many conditions. Excessive daytime sleepiness is a risk factor for involvement in traffic accidents [1], quality of life impairment, and impaired working capacity [2] and so has an important socioeconomic impact. In the general population up to 18% suffer from increased daytime sleepiness [2]. Common risk factors, such as chronic sleep deprivation, poor sleep hygiene, and female gender, have been shown to contribute to this burden [2]. Obstructive Sleep Apnea (OSA) is also a frequent cause of excessive daytime sleepiness and can even be defined by hypersomnolence if occurring together with disturbed respiration [3]. Furthermore various diseases, such as depression, obesity, periodic limb movement, and restless legs syndrome, have been shown to be associated with excessive daytime sleepiness [2]. In patients with suspected OSA, Chronic Obstructive Pulmonary Disease (COPD) is also a contributing factor to excessive daytime sleepiness [4]. Overlap syndrome as coexistence of OSA and COPD occurs in about 1% of the general population. Smoking and age are risk factors for both diseases. A higher BMI predisposes to OSA [5]. Also in COPD patients with higher BMI more respiratory events have been found. COPD affects airways and lungs and is characterized by progressive and not fully reversible airflow limitation and increased inflammatory response to gas and noxious particles [6]. Approximately one-third of current smokers have signs of COPD [7]. Its prevalence is estimated at 8–10% in the adult population with a higher percentage among males [8, 9]. COPD patients often suffer from additional comorbidities such as hypertension and depression [10].

COPD patients were found to suffer from shorter total sleep time (TST) and diminished sleep efficiency compared to healthy subjects [11]. Subjective measures such as Pittsburgh Sleep Quality Index confirm that COPD patients suffer from poor quality of sleep [12] with difficulties in falling asleep, delay of onset of sleep, frequent awakenings, use of hypnotics, and daytime sleepiness [11]. These disturbances can lead to reduced quality of life, anxiety or depression, and increased use of hypnotics [13]. Furthermore COPD patients showed altered patterns of physical activity (PA) and energy expenditure (EE) [14]. In non-COPD patients, a correlation between nocturnal EE and respiratory disturbances has been found [15]. Eventually, changes in nocturnal and diurnal EE and PA could also be related to daytime sleepiness.

A recent study of an Iranian COPD cohort compared daytime sleepiness and quality of sleep in COPD patients to a healthy control group. They found significantly more subjects suffering from excessive daytime sleepiness among COPD patients than among controls. Patients with moderate COPD seemed to be the most affected [16].

However, predictors of daytime sleepiness, especially the role of underlying sleep-related breathing disorder, still remain unclear.

Therefore, the present study aimed to evaluate potential associations of daytime sleepiness with disturbed sleep-related breathing and nocturnal activity in COPD patients.

## 2. Methods

### 2.1. Subjects

Ninety-one patients of the tertiary University Clinic Basel in Switzerland with COPD of Global Initiative for Chronic Obstructive Lung Disease (GOLD) grades 1–4 were recruited in a cross-sectional manner. The diagnosis of COPD was established by performing lung function during previous study participation and previous hospitalization or by the patient's general practitioner. Exacerbation during the last 6 weeks was an exclusion criterion. The present investigation was approved by the local ethics committee Ethikkommission beider Basel (EKBB, 163/11) and written informed consent was obtained from all patients prior to study entry.

### 2.2. Clinical Interview

In a clinical interview height and weight, need for oxygen, number of exacerbations in the previous 12 months, current medication, professional employment, average sleep duration during day and night, and smoking status were assessed. Body Mass Index (BMI) was calculated according to weight (kg)/(height)^2^ (m^2^). Exacerbation was defined as a worsening of the subject's condition in comparison to the stable state and beyond normal day-to-day variations, requiring additional treatment with oral or intravenous corticosteroids or antibiotics [17]. Patients were classified as current smokers, never-smokers, or ex-smokers (if they have stopped smoking more than one year ago) [18]. Comorbidities were assessed by consulting the hospital database and evaluating the patients' most recent physicians/discharge report. As reference for side effects of current medication, such as fatigue/sleepiness or sleep disturbances, the Swiss pharmaceutical compendium was consulted (https://www.compendium.ch/).

### 2.3. COPD Assessment

Airflow limitation and symptoms of COPD were assessed according to the GOLD guidelines [19]. The degree of airflow limitation was examined using spirometry with an Easyone Spirometer (ndd, Zurich, Switzerland). Symptoms were recorded with the COPD Assessment Test (CAT) and classified as mild or few symptoms if CAT was <10 and severe or many symptoms if CAT was ≥10 [20].

### 2.4. Sleep Evaluation, Energy Expenditure, and Physical Activity

In an outpatient setting, ApneaLink, a portable device, recorded pulse oximetry and nasal airflow measurements during one night in each patient [21]. The following parameters delivered by ApneaLink have been evaluated: Apnea Hypopnea Index (AHI), evaluation time of airflow, snoring events, and number of oxygen desaturations. Apnea was defined as a decrease in airflow by 80% of baseline for at least 10 seconds and a hypopnea was defined as a decrease in airflow by 50% to 80% for at least 10 seconds or as a decrease in airflow by 30% followed by an oxygen desaturation of at least 4% during the following 10 seconds. An oxygen desaturation was defined as a decrease in oxygen saturation of at least 4% [21–23].

The BodyMedia SenseWear Mini Armband is a wireless activity monitor with a three-axis accelerometer. Additionally, the multisensory device measures heat flux, skin temperature, and galvanic skin response [24]. Patients were instructed to wear the Armband for 7 consecutive days and nights. The following parameters have been evaluated: active EE, physical activity level (PAL), sleep duration, and number and duration of single sleep episodes [15]. For diurnal activity values, means were calculated over complete measurement days using daytime recordings. For nocturnal activity values, only one night, in which the ApneaLink was worn, has been analyzed. Detailed information about the analysis of the SenseWear Mini Armband data is described elsewhere [25].

### 2.5. Daytime Sleepiness and Quality of Sleep: Epworth Sleepiness Scale and Sinonasal Outcome Test-20

The German Version of the Epworth Sleepiness Scale (ESS) was used to assess subjects' general level of daytime sleepiness. A value ≥ 10 was considered to be pathological [16].

Furthermore we assessed the German Adapted Version of the Sinonasal Outcome Test-20 (SNOT-20), an instrument for measuring health-related quality of life (QOL) in patients with chronic rhinosinusitis with special regard to the subscore “general quality of life” comprising questions about sleep quality, depressive symptoms, and fatigue [26, 27].

### 2.6. Data Analysis

Data were analyzed using IBM SPSS 21. Descriptive analysis was performed and data distribution was analyzed with the Shapiro-Wilk test. Normally distributed data were analyzed with the Student *t*-test or one-way analysis of variance. If data were not normally distributed, Mann–Whitney test or Kruskal-Wallis test was applied. Categorical data were analyzed with Chi-square test or Fisher's exact test. *p* values < 0.05 were considered as statistically significant. Data are presented as mean ± standard deviation (SD) and median with interquartile range (IQR) dependent on data distribution. For some parameters (AHI, BMI, and ESS), which were not normally distributed, we also present mean values in order to facilitate comparison to other studies presenting mean ± SD only. Spearman correlations were calculated for nonparametric data and Pearson correlations for parametric data. Forced-entry multiple linear regression analysis was performed to identify potential predictors of ESS. Variables, which showed a significant bivariate correlation with ESS and primary outcome parameters of this study (EE during night and day and AHI), were included in the model. BMI was not included in the model because of its high collinearity with AHI (*r* = 0.613, *p* < 0.01).

Besides descriptive statistics for COPD assessment we analyzed bivariate correlation of CAT score as a parameter for severity of COPD with ESS and parameters of EE (active EE during day or night and metabolic equivalents (METs) for PAL measured with SenseWear).

Daytime sleepiness (ESS) was compared between COPD grades using Kruskal-Wallis test.

Association of ESS was correlated with parameters of sleep (number of awakenings, AHI, sleep efficiency, and TST) and parameters of energy expenditure (EE during day or night, METs, and active EE during day or night) were analyzed with bivariate correlations.

## 3. Results

### 3.1. Demographic and Anthropometric Data

**Figure 1:**
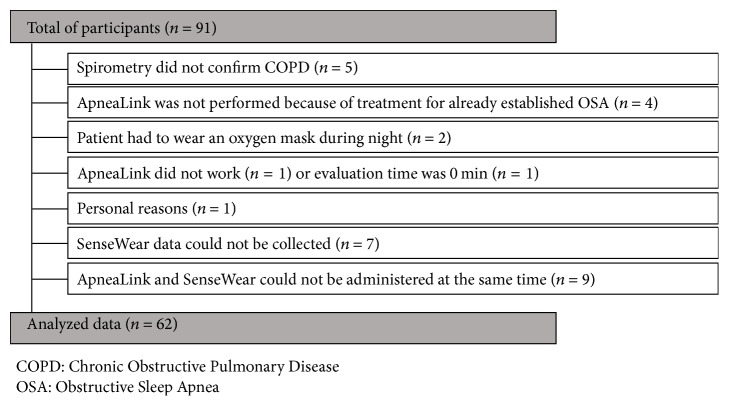
Reasons for exclusion/nonevaluation after recruitment.

Data of 91 participants were assessed and evaluated for enrollment into the study. Reasons for exclusion or nonevaluation are presented in Figure 1. Finally, 62 participants had a complete dataset and were considered for analysis. Mean age was 65 ± 9 years, ranging from 44 to 90 years. Mean BMI was 26 ± 5 kg/m^2^ with a range from 16 to 45 kg/m^2^. There was no difference between male and female patients regarding age, BMI, or smoking status.

Fourteen patients (23%) were in full- or part-time employment. Two of them had shift work, one once every two months and one 5 times a month (missing data in 4 subjects).

Patients excluded were comparable to the participants concerning age, BMI, smoking status, and severity of COPD.

Anthropometric data and demographic characteristics are presented in Table 1.

### 3.2. Comorbidities and Medication

At the time of examination, 7 patients (11%) had diabetes mellitus. Twenty-three patients (37%) were diagnosed with cardiovascular disease (CVD) and another 7 patients (11%) had depression. None of these comorbidities seemed to influence daytime sleepiness (data not shown). Nine patients (14.5%) did not take any medication with a side effect possibly influencing daytime sleepiness. Thus 85.5% of this cohort took at least one medication which possibly causes sleep disturbances or sleepiness. Nevertheless, neither ESS nor other subjective or objective parameters of sleep seemed to be altered by the intake of such medication.

### 3.3. COPD Assessment

Patients in this cohort predominantly suffered from mild to moderate COPD. Distribution across COPD grades and risk groups is presented in Table 1. As expected, lung function was worse in patients who suffered from more severe COPD symptoms (*r* = −0.444, *p* < 0.01). Younger patients were found to be more affected by severe COPD symptoms measured with CAT (*r* = −0.231, *p* < 0.05). Severity of COPD symptoms (CAT) correlated with increased daytime sleepiness (ESS) (*r* = 0.386, *p* < 0.01), SNOT-20 score (*r* = 0.398, *p* < 0.01) and ALQ subscore (*r* = 0.348, *p* < 0.01), reduced active EE (*r* = −0.309, *p* < 0.01), and PAL (*r* = −0.211, *p* < 0.05).

### 3.4. Sleep Parameters

The average self-reported sleep duration at night was 421 ± 96 min and during day 24 ± 38 min (missing data in 2 subjects). In objective measurement during one night TST of our patients was 355 min (IQR = 176; mean = 344 ± 109 min). Patients had a sleep efficiency of 88% (IQR = 15) and 8 sleep epochs (IQR = 8) with a median duration of 40 min (IQR = 45). Median AHI was 3.5 events/h (IQR = 7; mean = 6.6 ± 8.6 events/h). The number of awakenings was 7 (IQR = 6) and median wake time was 44 min (IQR = 60). Oxygen desaturations were observed in 39 patients (63%) and occurred 22 times per night (IQR = 45). Sleep parameters according to daytime sleepiness are listed in Table 2.

TST in the clinical interview and objective measurement with ApneaLink did not correlate with each other. AHI measured with ApneaLink and the number of sleep epochs measured with SenseWear correlated significantly (*r* = 0.255, *p* < 0.05). Compared to women, male patients more often had a pathologic AHI (*p* < 0.05) and a higher mean AHI (*p* < 0.05, Table 1). They also suffered more awakenings (*p* < 0.05) and lower sleep efficiency (*p* < 0.05).

### 3.5. Daytime Sleepiness

Eleven patients (18%) suffered from excessive daytime sleepiness with an ESS ≥ 10/24. Median ESS was 5.5 (IQR = 5) with a range from 0 to 19. ESS did correlate neither with parameters of disturbed sleep (AHI, oxygen desaturations, snoring events, and wake time) nor with EE during night and day. ESS did not differ between women and men. Characteristics of participants distinguished according to pathologic and normal daytime sleepiness are presented in Table 2.

There was a significant difference in daytime sleepiness between COPD risk groups A–D (*p* < 0.01), as risk group B showed the highest mean ESS of 7.8 ± 4.5 (median was 7.5, IQR = 5, and *p* < 0.01). In mild COPD (risk group A), no patient had a pathologic ESS and in severe COPD (risk groups C and D), no patient had a value of 0 points. Comparison of risk groups revealed that there were significantly more patients with pathologic ESS in risk group B than in risk group A (*p* < 0.01).

In bivariate analysis, ESS correlated inversely with age (*r* = −0.347, *p* < 0.01) and positively with CAT score (*r* = 0.386, *p* < 0.01). Furthermore ESS correlated positively with self-reported sleep duration during daytime (*r* = 0.408, *p* < 0.01) and negatively with sleep duration at night (*r* = −0.283, *p* < 0.05). Excessive daytime sleepiness also correlated with a higher SNOT-20 score (*r* = 0.261, *p* < 0.05) and a higher ALQ subscore (*r* = 0.268, *p* < 0.05). If patients were working or not did not seem to influence either daytime sleepiness or sleep behavior or sleep quality. Objective parameters of sleep, such as AHI, number of awakenings, sleep efficiency, and TST, showed no significant association with ESS. Increased nocturnal EE did not increase ESS and there was no correlation between ESS and active EE during daytime.

### 3.6. Multiple Regression Analysis

Forced-entry multiple linear regression analysis (Table 3) revealed that age, CAT, and AHI were independent predictors of ESS. The model explained in total 30% of the variance in ESS (adjusted *R* ^2^ = 0.299).

## 4. Discussion

Aiming to evaluate possible predictors of excessive daytime sleepiness in a cohort of mainly mild to moderate COPD patients, this study provides evidence that impaired nocturnal breathing may increase daytime sleepiness in COPD.

### 4.1. Prevalence and Severity of Daytime Sleepiness

In the present cohort, mean ESS score was within the normal range and only 18% of study participants suffered from excessive daytime sleepiness. The severity of daytime sleepiness in the examined COPD patients was comparable to previous findings in cohorts without COPD.

Zohal et al. compared 120 middle-aged patients with mild to severe COPD with an age- and gender-matched control group and found a prevalence of excessive daytime sleepiness (ESS ≥ 10) of 32% in COPD patients. In their study, only 15% of the healthy controls suffered from excessive daytime sleepiness [16]. However, more males and more patients with severe COPD (FEV1 < 50% predicted) were evaluated compared to our study, which may have influenced the results, as this has been shown to be related to lower quality of sleep and more severe daytime sleepiness [16]. Also in the present study, male patients suffered from significantly lower quality of sleep measured as lower sleep efficiency, more awakenings, and higher number of sleep epochs of shorter duration.

On the other hand, in the cohort of the Sleep Heart Health Study, a mean ESS of 7.8 (95% CI 7.41, 8.14) was found in patients with mostly mild to moderate COPD. There was no significant difference of ESS score in patients with COPD compared to a healthy control group [9].

### 4.2. Comorbidities and Medication

In patients with CVD, excessive daytime sleepiness was not more frequent than in patients without CVD and mean ESS did not differ significantly. However, patients with CVD were found to have a significantly lower quality of sleep, measured by lower sleep efficiency and more awakenings. This is in accordance with previous studies indicating a certain association between combined sleep problems and arterial hypertension [28]. In other studies diabetes mellitus and depression have been described among others to be determinants of daytime sleepiness [4]. In the present study, ESS showed no significant association with diabetes or depression. Patients with depression or diabetes were found to have only a slightly increased ESS compared to patients who did not suffer from any of these comorbidities. Therefore we did not exclude these patients. However only 11% (*n* = 7) suffered from depression or diabetes, and thus our results need to be interpreted with caution. Furthermore most of our patients were under medication which could cause sleeping disorder or fatigue. Regarding the fact that in our patients 85% had at least one medication which can have an influence on sleep or sleepiness we could not consider these possible side effects as exclusion criterion. We though assume that in a real setting most patients are under medication, which can cause fatigue. However, parameters of sleep did not differ between patients with or without medications. To our knowledge only 2 patients had shift work during the study period. Thus, we could not get representative results concerning the role of night work in daytime sleepiness. However, daytime sleepiness was not significantly higher in patients who were employed.

### 4.3. Daytime Sleepiness, COPD Symptoms, and Age

All subjectively assessed variables CAT, SNOT-20 (as well as ALQ subscore), and ESS correlated inversely with age. This could be due to a different perception and estimation of sleepiness. Younger patients with COPD might feel more impaired by their disease than the elderly. For example, older individuals may have adapted to sleepiness, whereas younger patients may find this limiting, because their peers are fitter [25]. Retired people may also have more control on how to spend the day. This was confirmed by previous studies showing that different work demands influenced daytime sleepiness [29]. Furthermore, in the present study, age was not distributed equally across COPD risk groups. Patients with moderate COPD (risk group B) were younger than patients with mild COPD (risk group A), which could have led to this inverse association. This might in addition have influenced the present results, as risk groups are partly defined by the CAT, which correlates with the ESS.

A significant correlation between ESS and CAT could be expected since CAT also includes items concerning sleep and subjective level of energy, which both correlated with ESS.

### 4.4. Daytime Sleepiness and Sleep Parameters

The COPD cohort of the Sleep Heart Health Study had a similar TST as our patients. In comparison to the healthy control group, COPD patients had a shorter TST. Nevertheless, daytime sleepiness did not differ between COPD patients and healthy controls [9]. This is consistent with our results where TST did not show any correlation with daytime sleepiness.

Self-reported sleep time and objective measurement were not coherent. Possible reasons could be that in the clinical interview patients indicated an average duration, while only one night was objectively measured. On the other hand patients probably overestimated real sleep duration by not noticing brief awakenings.

### 4.5. Daytime Sleepiness and Disturbed Nocturnal Breathing

In the present study, no significant difference in daytime sleepiness was found between patients with or without nocturnal breathing disorder. However, we detected a slightly higher ESS in patients with an AHI ≥ 5 than in patients with normal nocturnal breathing. This tendency increases only minimally when choosing a cut-point of AHI ≥ 10 events/h.

This finding is not in accordance with previous studies which found a significant correlation between excessive daytime sleepiness and sleep-related breathing disorder in COPD patients [9]. Lewis et al. compared daytime sleepiness in patients with and without nocturnal oxygen desaturations and did not find any difference [30]. The present study does confirm this result. There were no significant differences in ESS between desaturators and nondesaturators or any significant correlations between the number of nocturnal oxygen desaturations and ESS score. Other studies found nonapneic desaturations in 11% of COPD patients, which is similar to 14% detected in our cohort.

Multiple linear regression analysis revealed that AHI was an independent predictor of daytime sleepiness measured with the ESS in COPD patients. This is consistent with Koutsourelakis et al., who identified AHI as one of the most important predictors of ESS in a population with high risk for OSA [4]. After AHI, diabetes mellitus, and depression, they found COPD, increased BMI, CVD, and alcohol to independently influence daytime sleepiness [4]. Reasons for a dissociation between ESS and sleep quality as previously described may be cultural norms, recall bias, not wishing to acknowledge sleepiness or a state of being alert caused by medication, sympathetic hyperstimulation, or anxiety [31]. Besides, Urschitz et al. examined children with subjective and objective methods regarding sleepiness and found no correlation between the two results, as if they were different measurements [32]. Nevertheless, subjective measurements are sensitive and valid indicators of sleepiness [33].

### 4.6. Limitations of ESS

Whether the ESS sufficiently reflects nocturnal symptoms is still controversial. Fong et al. concluded that the ESS was not sensitive enough to reflect the degree of daytime sleepiness referring to the severity of OSA [34]. According to this Scharf et al. examined a cohort of COPD patients and found no difference in daytime sleepiness or quality of life between overlap patients and patients with COPD alone. Insomnia without excessive daytime sleepiness was the primary sleep complaint in their cohort [31]. One possibility is that, being a chronic disease, COPD itself influences the susceptibility to sleepiness and could therefore independently impact the ESS score, as it has already been suggested by previous studies [4]. Therefore further investigations using objective measurement tools to assess daytime sleepiness, such as the Multiple Sleep Latency Test (MSLT), may be required in this subject area [34].

### 4.7. Study Limitations

Our study has some limitations. Polysomnography is the gold standard method for sleep evaluation. However, because of its high technical requirements and financial costs, two portable, validated devices were used instead to measure sleep parameters [15, 22].

The SenseWear Mini Armband was found to be reliable in estimating sleep but showed a low specificity in determining wakefulness. Sleep episodes seem to be well captured. But as sleep tends to be overestimated, it is recommended to use the Armband together with other devices [15]. The results of the ApneaLink device (AHI) and SenseWear Mini Armband (number of sleep epochs and awakenings) correlated with each other.

AHI as assessed by the ApneaLink was calculated based on the evaluation time of the device. Therefore, AHI is underestimated [35] and the prevalence of disturbed sleep might even be higher in this cohort. In a further step, the application of SenseWear data to calculate AHI with the provided TST could lead to a more accurate value of AHI.

Furthermore, a selection bias could have occurred in this study, as patients were recruited according to former hospitalization and former participation in previous COPD studies. Therefore, patients with very mild or recently diagnosed COPD were unlikely to be included. On the other hand, only a few patients with severe COPD agreed to participate in this study. In consequence, the present results may only be representative for patients with mild to moderate COPD. Further investigation is required to confirm our results for patients with newly diagnosed or very severe COPD.

## 5. Conclusions

The present findings suggest that nocturnal breathing disorders may be associated with daytime sleepiness in COPD patients. Furthermore this study provides evidence for an association of daytime sleepiness with age and COPD-related quality of life impairment.

## Figures and Tables

**Table 1:** Demographic and anthropometric data.

Variables	Female (*n* = 28)	Male (*n* = 34)	Total (*n* = 62)
*Mean age *(*a*)	64 ± 9	66 ± 9	**65 **±** 9**

*Mean BMI (kg/m* ^*2*^)	24 ± 4	27 ± 6	**26 **±** 5**

*Smoking status*			
Never-smokers	0	1 (2.9%)	**1 (1.6%)**
Ex-smokers	11 (39.3%)	20 (58.8%)	**31 (50.0%)**
Current smokers	17 (60.7%)	13 (38.2%)	**30 (48.4%)**
Pack years	40 ± 30	46 ± 32	**43 **±** 31**

*COPD grade*			
Grade 1	12 (42.9%)	8 (23.5%)	**20 (32.3%)**
Grade 2	14 (50.0%)	16 (47.1%)	**30 (48.4%)**
Grade 3	2 (7.1%)	6 (17.6%)	**8 (12.9%)**
Grade 4	0	4 (11.8%)	**4 (6.5%)**

*COPD risk group*			
Risk group A	11 (39.3%)	11 (32.4%)	**22 (35.4%)**
Risk group B	15 (53.6%)	13 (38.2%)	**28 (45.2%)**
Risk groups C and D	2 (7.1%)	10 (29.4%)	**12 (19.4%)**

*Sleep-related breathing*			
Mean AHI	3.8 ± 4.3	8.8 ± 10.5	**6.6 **±** 8.6**
Pathologic (AHI ≥ 5)	10 (35.7%)	21 (61.8%)	**31 (50%)**
Nonpathologic (AHI < 5)	18 (64.3%)	13 (38.2%)	**31 (50%)**

*Daytime sleepiness*			
Mean ESS	6 ± 4	6 ± 4	**6 **±** 4**
Excessive (ESS ≥ 10)	5 (17.9%)	6 (17.6%)	**11 (17.7%)**
Normal (ESS < 10)	23 (82.1%)	28 (82.4%)	**51 (82.3%)**

AHI: Apnea Hypopnea Index, BMI: Body Mass Index, COPD: Chronic Obstructive Pulmonary Disease, ESS: Epworth Sleepiness Scale, and OSA: Obstructive Sleep Apnea.

**Table 2:** Daytime sleepiness.

Variables	Excessive daytime sleepiness ESS ≥ 10 (*n* = 11)	Normal daytime sleepiness ESS < 10 (*n* = 51)	Sig.
Gender			0.983
Male	6	28	
Female	5	23	
Age	59 y (±7)	67 y (±9)	0.009
BMI	27 kg/m^2^ (±8)	25 kg/m^2^ (±5)	0.912

*COPD*			
CAT	20 (IQR = 5)	12 (IQR = 9)	0.000
FEV1	70% (IQR = 23)	75% (IQR = 35)	0.804
COPD grades			0.624
Grade I	3 (27.3%)	17 (33.3%)	
Grade II	7 (63.4%)	23 (45.1%)	
Grade III	1 (9.1%)	7 (13.7%)	
Grade IV	0	4 (7.8%)	
COPD risk group			0.003
Risk group A	0	22 (43.1%)	
Risk group B	10 (90.9%)	18 (35.3%)	
Risk group C/D	1 (9.1%)	11 (21.6%)	

*Sleep-related breathing *			
Mean AHI	8 events/h (±12)	6 events/h (±8)	0.817
Median AHI	3.0 (IQR = 12)	4.0 (IQR = 7)	1.000
AHI			0.830
Pathologic (AHI ≥ 5)	5 (45.5%)	25 (49.0%)	
Nonpathologic (AHI < 5)	6 (54.5%)	26 (51.0%)	
Median oxygen desaturations	26 (IQR = 146)	21 (IQR = 44)	0.985
Snoring events	781 (IQR = 909)	647 (IQR = 1648)	0.905

*Energy expenditure*			
EE at night	1925 J (IQR = 1431)	2248 J (IQR = 824)	0.537
Active EE during day	1042 J (IQR = 1256)	1402 J (IQR = 2126)	0.362
MET at night	0.9 (IQR = 0.2)	0.9 (IQR = 0.1)	0.977

*Sleep *			
Sleep episodes	8 (IQR = 6)	8 (IQR = 7)	0.644
Total sleep time	306 min (IQR = 180)	356 (IQR = 174)	0.761
Wake time	31 min (IQR = 63)	50 min (IQR = 66)	0.261
Sleep efficiency	90% (IQR = 10)	87% (IQR = 15)	0.433

*Comorbidities*			
Cardiovascular disease	4 (36.4%)	19 (37.3%)	0.956
Diabetes mellitus	3 (27.3%)	4 (7.8%)	0.065
Depression	2 (18.2%)	5 (10.0%)	0.426

*Daytime sleepiness*			
Mean ESS	12 pts (±3)	5 pts (±2)	0.000
Median ESS	11 (IQR = 6)	5 (IQR = 4)	0.001

*SNOT-20*			
SNOT-20 (total)	24 (IQR = 25)	16 (IQR = 18)	0.126
ALQ subscore	14 (IQR = 17)	7 (IQR = 9)	0.104

AHI: Apnea Hypopnea Index, BMI: Body Mass Index, COPD: Chronic Obstructive Pulmonary Disease, EE: energy expenditure, ESS: Epworth Sleepiness Scale, FEV1: Forced Expiratory Volume in 1 second, MET: metabolic equivalent, OSA: Obstructive Sleep Apnea, and SNOT: Sinonasal Outcome Test.

**Table 3:** Forced-entry multiple linear regression, outcome variable = ESS (pts) (*n* = 62).

Model	*B*	SE *B*	*β*	Sig.
Age	−0.105	0.48	−0.254	**0.035**
CAT	0.213	0.068	0.380	**0.003**
Nocturnal EE	−0.001	0.002	−0.061	0.627
Active EE during daytime	−0.001	0.001	−0.056	0.639
AHI	0.129	0.057	0.287	**0.027**

AHI: Apnea Hypopnea Index, *B*: regression coefficient *B*, CAT: COPD Assessment Test, EE: energy expenditure, and SE: Standard Error.

## Additional Points

*Clinical Implication*. Previous studies have shown that particularly patients with severe COPD and OSA benefit from CPAP therapy regarding mortality [36]. We suppose it may be beneficial for patients with mild to moderate COPD to be screened for daytime sleepiness and nocturnal breathing disorder. A further therapy for suitable patients may also improve their quality of life.
